# The Impact of Preoperative Risk on the Association between Hypotension and Mortality after Cardiac Surgery: An Observational Study

**DOI:** 10.3390/jcm9072057

**Published:** 2020-06-30

**Authors:** Vanja Ristovic, Sophie de Roock, Thierry G. Mesana, Sean van Diepen, Louise Y. Sun

**Affiliations:** 1 Division of Cardiac Anesthesiology, University of Ottawa Heart Institute, Ottawa, ON K1Y 4W7, Canada; vristovic@toh.ca; 2 Division of Cardiology, University of Ottawa Heart Institute, Ottawa, ON K1Y 4W7, Canada; sderoock@ottawaheart.ca; 3 Division of Cardiac Surgery, University of Ottawa Heart Institute, Ottawa, ON K1Y 4W7, Canada; tmesana@ottawaheart.ca; 4 Cardiocore Big Data Research Unit, University of Ottawa Heart Institute, Ottawa, ON K1Y 4W7, Canada; 5 Department of Critical Care and Division of Cardiology, Department of Medicine, University of Alberta, Edmonton, AB T6G 2R7, Canada; sv9@ualberta.ca; 6 School of Epidemiology and Public Health, University of Ottawa, Ottawa, ON K1Y 4W7, Canada

**Keywords:** cardiac surgery, hemodynamic monitoring, hypotension, mortality, pre-operative risk

## Abstract

Background: Despite steady improvements in cardiac surgery-related outcomes, our understanding of the physiologic mechanisms leading to perioperative mortality remains incomplete. Intraoperative hypotension is an important risk factor for mortality after noncardiac surgery but remains relatively unexplored in the context of cardiac surgery. We examined whether the association between intraoperative hypotension and in-hospital mortality varied by patient and procedure characteristics, as defined by the validated Cardiac Anesthesia Risk Evaluation (CARE) mortality risk score. Methods: We conducted a retrospective cohort study of consecutive adult patients who underwent cardiac surgery requiring cardiopulmonary bypass (CPB) from November 2009–March 2015. Those who underwent off-pump, thoracic aorta, transplant and ventricular assist device procedures were excluded. The primary outcome was in-hospital mortality. Hypotension was categorized by mean arterial pressure (MAP) of <55 and between 55–64 mmHg before, during and after CPB. The relationship between hypotension and death was modeled using multivariable logistic regression in the intermediate and high-risk groups. Results: Among 6627 included patients, 131 (2%) died in-hospital. In-hospital mortality in patients with CARE scores of 1, 2, 3, 4 and 5 was 0 (0%), 7 (0.3%), 35 (1.3%), 41 (4.6%) and 48 (13.6%), respectively. In the intermediate-risk group (CARE = 3–4), MAP < 65 mmHg post-CPB was associated with increased odds of death in a dose-dependent fashion (adjusted OR 1.30, 95% CI 1.13–1.49, per 10 min exposure to MAP < 55 mmHg, *p* = 0.002; adjusted OR 1.18 [1.07–1.30] per 10 min exposure to MAP 55–64 mmHg, *p* = 0.001). We did not observe an association between hypotension and mortality in the high-risk group (CARE = 5). Conclusions: Post-CPB hypotension is a potentially modifiable risk factor for mortality in intermediate-risk patients. Our findings provide impetus for clinical trials to determine if hemodynamic goal-directed therapies could improve survival in these patients.

## 1. Introduction

Intraoperative hypotension is an established risk factor for in-hospital and one-year mortality after noncardiac procedures, and cardiovascular and renal-related complications after both cardiac and noncardiac surgery [1,2,3,4,5,6]. A recent multicenter study of major noncardiac surgery suggests that the association between intraoperative hypotension and postoperative risk of acute kidney injury (AKI) varies by baseline patient and procedure risk, such that AKI was not associated with hypotension in low-risk patients, whereas an association was found with severe hypotension in the medium-risk group, and with milder degrees of hypotension in the highest-risk group [7,8]. Studies in patients undergoing major cardiac surgery also reported an increase in the risk of 30-day mortality in low-risk patients who exhibited perioperative systolic blood pressure variation (which included hypotensive episodes) compared to high-risk patients [9]. Despite this evidence, the interaction between hypotension and baseline patient disease burden has not been clearly defined in cardiac surgery patients. We hypothesized that the association between mean arterial pressure (MAP) < 65 mmHg and mortality is accentuated in patients with higher baseline risk. We examined whether the association between intraoperative hypotension and in-hospital mortality varied across different baseline risk profiles, as defined by individual patient and procedure characteristics. 

## 2. Experimental Section

### 2.1. Design and Selection Criteria

The study protocol was approved by the University of Ottawa Heart Institute Research Ethics Board, which waived the requirement for individual patient consent. We conducted a study of consecutive adult patients who underwent major cardiac surgery requiring cardiopulmonary bypass (CPB) between 1 November 2009 and 3 March 2015 at the University of Ottawa Heart Institute. Patients who underwent off-pump or thoracic aortic procedures, as well as cardiac transplantation and insertion of ventricular assist devices, were excluded. The reporting of this study adhered to the Strengthening the Reporting of Observational Studies in Epidemiology guidelines. The data was analysed using a pre-specified primary outcome and statistical analysis plan.

### 2.2. Data Sources

We performed an analysis of prospectively collected data from Cardiocore. Cardiocore is a multimodular data repository that captures detailed demographics, comorbidities, physiologic and procedural details, continuous hemodynamic measurements, perioperative management and outcomes for all patients who undergo cardiac procedures at the University of Ottawa Heart Institute, a university-affiliated tertiary cardiac care referral center that performs the full scope of cardiac operations. It is managed by a multidisciplinary committee and undergoes regularly scheduled quality assurance audits [3,4].

Cardiocore also captures intraoperative invasive blood pressure measurements, which were recorded every 15 s from an electronic patient record (CompuRecord, Philips Medical Systems, Eindhoven, The Netherlands). Any artifacts were removed by using an automated algorithm as previously described [3,4]. Specifically, because there were up to four recorded MAP values per minute, the median of these values was selected for the analysis. Time periods corresponding to absent (no MAP readings) or aberrant MAP values (an isolated MAP value that differed more than 50% from both preceding and subsequent values) were deleted. This approach effectively removed artifacts in invasive MAP values related to manipulation of the arterial line including blood sampling, clamping, flushing, and zeroing [3]. In the event when arterial tracings were dampened, femoral or direct aortic pressure monitoring was provided by the surgeon via a three-way stopcock, connected to the original arterial line transducer. In these cases, the electronic record is automatically populated by the new arterial pressure recordings. MAP readings were analyzed from the onset of end-tidal carbon dioxide (i.e., induction) until the last end-tidal carbon dioxide reading (i.e., the conclusion of anesthesia and transfer of patient from the operating room to the intensive care unit). We routinely place arterial lines before induction at our institution, with the exception of critical emergencies such as aortic rupture, which were excluded from this analysis. Intraoperative hemodynamic data was processed by using R [version 3.2.1; https://cran.rproject.org/bin/windows/base/old/3.2.1/; accessed on 1 June 2015].

### 2.3. Outcome and Exposure

The primary outcome was all-cause mortality during the index surgical admission. Hypotension was categorized as MAP of <55 and between 55–64 mmHg before, during and after CPB. The selected MAP thresholds were based on thresholds shown to be associated with complications or death in recent studies of hypotension during cardiac and noncardiac surgery [2,4,5,9,10].

Previous studies in cardiac surgery patients have been focused on blood pressure management during the CPB period [9,11,12,13]. We were motivated to study the periods before, during, and after CPB in our analyses due to their physiologic distinctness. During CPB, MAP is driven by the pump flow and systemic vascular resistance. Although MAP before and after CPB are both driven by systemic vascular resistance and intrinsic cardiac function, adequate return to pulsatile flow after CPB is also influenced by factors such as CPB duration, the degree of myocardial preservation, and air embolism.

### 2.4. Stratification of Baseline Risk

We used the Cardiac Anesthesia Risk Evaluation (CARE) score to classify baseline patient and procedure risk [14]. CARE is a validated mortality risk score that integrates the severity of cardiac disease, co-existing medical problems including degree of disease control, and complexity of cardiac surgery to be performed. It represents a “clinician’s gestalt” of baseline patient and procedure risk and has been shown to perform as well as other multifactorial risk indexes such as the EuroScore for predicting outcome in cardiac surgery [15]. A CARE score of 1–2 depicts low patient and procedure risk, 3–4 depicts intermediate-risk, and a score of 5 depicts high-risk (C-statistic = 0.80) [14].

### 2.5. Statistical Analysis

We began by conducting our analysis in the overall cohort. Continuous variables were analyzed by using the Wilcoxon rank sum test and presented as median (interquartile range). Categorical variables were analyzed by using chi-square test and presented as number (proportion).

The possible association between hypotension and in-hospital death was examined by using logistic regression with adjustment for a priori selected risk factors, including key components of the Society of Thoracic Surgeons score [16] and the EuroScore II [17]. These risk factors are detailed in Table 1 and include age, sex, left ventricular ejection fraction, heart failure, hypertension, peripheral arterial disease, atrial fibrillation, diabetes, renal insufficiency, emergent surgery, preoperative cardiogenic shock, type of surgery, re-operative status, CPB duration, nadir hematocrit during CPB, intraoperative blood transfusion, and the requirement for inotrope or vasopressors. The definitions for these covariates are provided in Table A1.

We examined for any modification of the effect of CARE score (categorized as 1–2, 3–4 and 5) on the association between MAP < 55 mmHg and death by simultaneously adding the following interaction terms to the overall multivariable model: CARE score * pre-CPB MAP < 55, CARE score * bypass MAP < 55, CARE score * post-CPB MAP < 55. Similarly, we assessed the overall model with the simultaneous addition of CARE score * pre-CPB MAP between 55–64, CARE score * bypass MAP between 55–64, and CARE score * post-CPB MAP between 55–64. As effect modification was present for both MAP thresholds during the post bypass period, we proceeded to stratify our analysis by low, intermediate and high baseline risk as defined by these CARE score categories.

Post-hoc, we examined the durations of hypotension in patients who did and did not develop major postoperative complications. These complications (stroke, new onset renal replacement therapy, and the composite of these endpoints) have established associations with postoperative mortality and are used to assess the robustness of our findings.

We used odds ratios (OR) and associated 95% confidence intervals (CI) to demonstrate the measure of association. We defined a minimum clinically meaningful effect as an OR of more than 1.05 per 10 min of hypotension, and more than 1.5 for other covariates [3,4]. As two different MAP thresholds were tested, we used the Bonferroni method to correct for multiple testing, with statistical significance defined by a two-tailed *p* < 0.025. All analyses were conducted using SAS version 9.4 (SAS Institute, Cary, NC, USA).

### 2.6. Missing Data

Main outcome and exposure variables were complete for all included subjects. Left ventricular ejection fraction was imputed using the group mean for 82 (1.3%) patients. Weight and height were imputed with the group mean for 4 and 5 patients, respectively. The proportion of absent and artifactual MAP values removed was less than 1% of the total.

## 3. Results

Among the 6627 patients included in this study, 131 (2.0%) died in-hospital. The baseline characteristics of the overall cohort is summarized in Supplemental Table S1, and the multivariable predictors of mortality in Supplemental Table S2. Out of those deceased, none were from the lowest-risk group (*n* = 397) and 7 (0.3%, *n* = 2363) had a CARE score of 2. Conversely, in-hospital mortality occurred in 76 (2.2%) of the 3513 intermediate-risk patients (CARE = 3–4) and 48 (13.6%) of the 354 high-risk patients (CARE = 5). 

Baseline risk modified the effect of MAP < 55 mmHg post-CPB on mortality (interaction *p*-value = 0.015) but not at the same MAP threshold before (interaction *p*-value = 0.127) or during CPB (interaction *p*-value = 0.510). Similarly, baseline risk modified the effect of post-CPB MAP between 55–64 mmHg on mortality (interaction *p*-value = 0.011), but not at the same MAP threshold before (interaction *p*-value=0.374) or during CPB (interaction *p*-value=0.152). 

In the intermediate-risk group, those who died were more frequently older, had pre-existing hypertension, heart failure, peripheral arterial disease, renal insufficiency, anemia, to present in cardiogenic shock, undergo emergent surgery with longer bypass duration, have a lower nadir hematocrit while on CPB, to require intraoperative transfusion and inotropic and vasopressor support (Table 1). In contrast, those who died in the high-risk group were more likely to have renal insufficiency, prolonged bypass duration, have a lower nadir hematocrit while on CPB, to require intraoperative transfusion and vasopressor support before CPB (Table 2). 

### Association between Intraoperative Hypotension and In-Hospital Mortality

Compared to the survivors, those who died in the intermediate-risk group experienced longer durations of MAP < 65 mmHg before, during and after CPB (Table 3). Patients in the intermediate-risk group who later suffered from stroke or required new onset renal replacement therapy also experienced longer durations of MAP < 65 mmHg throughout surgery (Supplemental Table S3a).

In the high-risk group, there were no statistically significant differences in the durations of hypotension experienced by survivors and non-survivors (Table 4). Of note, patients in this risk group who required new onset renal replacement therapy also experienced longer durations of MAP < 65 mmHg after CPB (Supplemental Table S3b).

Table 5 demonstrates the association between hypotension and death, stratified by baseline risk. In the intermediate-risk group, post-CPB hypotension was associated with increased odds of death in a dose-dependent manner. Specifically, each 10 min epoch of MAP < 55 mmHg post-CPB was associated with a 30% increased odds of death (adjusted OR 1.30; 95% CI, 1.13–1.49; *p* = 0.002), and each 10 min epoch of MAP between 55–64 mmHg post-CPB was associated with an 18% increased odds of death (adjusted OR 1.18; 95% CI, 1.07–1.30; *p* = 0.001). There was no statistically significant association between intraoperative hypotension and death in the high-risk group. Supplemental Table S4 summarizes the association between hypotension and death in the low-risk group. 

The predictors of in-hospital mortality, stratified by preoperative risk, are summarized in Supplemental Table S5. The multivariable models were accurate and reliable for both groups (c-statistic = 0.90, Hosmer-Lemeshow *p*-value = 0.72 for the intermediate-risk model; c-statistic = 0.81, Hosmer-Lemeshow *p*-value = 0.88 for the high-risk model). In addition to post-CPB hypotension in the intermediate-risk group, older age, a history of hypertension, peripheral arterial disease, renal insufficiency, prolonged CPB duration, transfusion of >4 units of red blood cells, and new onset atrial fibrillation were also independently associated with mortality in-hospital (Supplemental Table S5a). In the high-risk group, pre-existing hypertension, renal insufficiency, >4 units of red blood cell transfusion and requirement for inotropic support post-CPB were independently associated with in-hospital mortality (Supplemental Table S5b).

## 4. Discussion

In intermediate-risk cardiac surgery patients, we found a clinically significant association between in-hospital mortality and MAP < 65 mmHg post-CPB in a dose-dependent fashion. Specifically in this group, each additional 10 min of MAP < 55 mmHg post-CPB was associated with a 30% increase in the odds of death (adjusted OR 1.30; 95% CI, 1.13–1.49; *p* = 0.002), and each additional 10 min of MAP between 55–64 mmHg post-CPB was associated with an 18% increase in the odds of death (adjusted OR 1.18; 95% CI, 1.07–1.30; *p* = 0.001). We however did not observe an association between intraoperative hypotension and death in the high-risk group.

Intraoperative hypotension has recently been established as an important risk factor for death, stroke, acute myocardial and kidney injury after cardiac and noncardiac surgery [2,3,4,5,7,9]. In the setting of cardiac surgery, previous large observational studies from our group found a higher rate of stroke in patients who experienced MAP < 65 mmHg for > 10 min during CPB, and a higher rate of renal replacement therapy in patients who experienced MAP < 65 mmHg for > 10 min post-CPB. On the other hand, a randomized controlled trial (RCT; N = 198) comparing the effect of maintaining higher (70–80 mmHg) vs. lower (40–50 mmHg) MAP targets during CPB using norepinephrine infusion, reported no difference in the number and size of new cerebral infarcts [18]. This same group of authors also reported in a substudy of a RCT (N = 36), that sublingual microcirculatory flow was no different in patients whose MAP was maintained in the 70–80 mmHg vs. 40–50 mmHg range during CPB [19]. In addition, a recent RCT (N = 199) reported that maintenance of MAP during CPB at the lower limit of the individual’s cerebral autoregulation reduced the incidence of delirium after cardiac surgery [20].

These previous studies have largely focused on the modeling of risk in generalized patient cohorts, rather than personalizing hemodynamic goals through a risk-stratified approach. The novelty of the present study resides in its individualized definition of critical MAP thresholds in accordance to baseline risk, during all physiologically distinct phases of cardiac surgery. Our findings suggest that post-CPB hypotension may be a modifiable risk factor in intermediate-risk patients (CARE mortality risk score of 3–4) but not in those at high-risk (CARE score of 5). Importantly, we highlight that intermediate-risk patients with features such as advanced age, pre-existing hypertension, peripheral arterial disease, renal insufficiency and prolonged bypass duration may benefit most from targeted hemodynamic management in the post-CPB period.

Our findings are overall in keeping with the literature. A recent multicenter study in the context of major noncardiac surgery suggests that the association of intraoperative hypotension and postoperative risk of acute kidney injury varied by baseline patient and procedure risk, such that acute kidney injury was not associated with hypotension in low-risk patients, whereas an association was found with severe hypotension (MAP < 50 mmHg) in the medium-risk group, and with milder degrees of hypotension (MAP between 55–59 mmHg) in the highest-risk group [7,8]. Aronson et al. took a similarly risk-stratified approach to study the impact of systolic blood pressure (SBP) variability in patients undergoing cardiac surgery and observed a higher rate of 30-day mortality in patients who had intraoperative SBP variability outside of a range of 75 to 135 mmHg in a dose-dependent fashion [11]. Of note from this study, low-risk patients had a 2.5-fold increase in the rate of death after being exposed to SBP variability, and high-risk patients a 1.4-fold increase in the rate of death. We found a dose-dependent relationship between post-CPB MAP < 65 mmHg and mortality in intermediate-risk patients but did not observe such an association in high-risk patients. The latter could in part be explained by the higher burden of comorbidities, as well as procedural complexity, acuity of disease presentation, and the higher frequency of pre-existing myocardial dysfunction and postoperative complications such as stroke and renal replacement therapy in the high-risk group as demonstrated in our post hoc analyses. These factors interact to elevate the risk of death regardless of perfusion pressure. In our study, both survivors and non-survivors from the high-risk group had similar burdens of most baseline risk factors and experienced similar durations of hypotension. Therefore, the lack of statistical power in this small subgroup may have in part contributed to the lack of statistical significance observed in the association between hypotension and mortality.

The post-CPB period is one of extreme physiologic stress secondary to tissue hypoxia, catecholamine surges, heightened inflammatory states, and decreased vasomotor reactivity [21,22]. This physiologic disadvantage is often further exacerbated in the presence of low cardiac output syndrome. Both extracorporeal contact of red blood cells with artificial surfaces [23] and the resulting mechanical trauma also trigger a significant inflammatory response, which may also worsen the degree of end organ injury, especially in the presence of post-CPB hypotension [24].

Hypotension is common in the operative setting. In fact, up to 44% of patients undergoing major noncardiac surgery [8] and 87% of patients undergoing cardiac surgery [3] experience at least one episode of MAP < 65 mmHg for ≥10 min. Vigilant intraoperative monitoring incorporating all routinely available data (i.e., MAP, pulse rate and contour, end tidal CO_2_), and proactive treatment addressing the underlying cause (e.g., hypovolemic, cardiogenic vs. distributive), are key to restoring pressure and flow. Given our findings, older intermediate-risk patients with pre-existing hypertension, peripheral arterial disease and renal insufficiency may benefit most from comprehensive approaches to blood pressure management.

### Limitations

Our study has several limitations. First, this is a single-center study. Further studies are needed to confirm the generalizability of our findings. Second, we are limited by the inherent biases of the cohort study design, such as residual confounding despite careful risk adjustment. Prospective randomized trials are needed to determine the impact of hypotension relative to associated factors such as low flow or hypovolemic states, as well as the management of these states. Third, we were unable to explore within the current study design the pressure-flow relationship, dosages of vasopressors and inotropes used, nor whether treatment of intraoperative hypotension could improve patient outcome.

## 5. Conclusions

In conclusion, we observed a higher risk of in-hospital mortality in intermediate-risk patients when MAP fell below 65 mmHg for more than 10 min after CPB. Specifically, each 10-min epoch of MAP < 55 mmHg post-CPB was associated with a 30% increased odds of death, while each 10-min epoch between 55–64 mmHg was associated with a 18% increased odds of death. Intraoperative hypotension was not independently associated with an increased risk of death in high-risk patients. Our findings highlight the importance of tailoring post-CPB MAP management according to baseline risk. Prospective studies are needed to evaluate the effectiveness of comprehensive hemodynamic assessment and management strategies in reducing perioperative deaths in intermediate-risk cardiac surgery patients.

## Figures and Tables

**Table 1:** Baseline and perioperative characteristics of intermediate-risk patients, as defined by a Cardiac Anesthesia Risk Evaluation (CARE) score of 3–4.

Variable	Not Deceased	Deceased	*p*-Value
	(*n* = 3437)	(*n* = 76)	
Demographic			
Age, median (IQR), year	68 (60–76)	78 (69–81)	<0.0001
Female sex, *n* (%)	1049 (30.5)	27 (35.5)	0.349
Medical history, *n* (%)			
Hypertension	2579 (75.0)	72 (94.7)	<0.0001
LVEF < 35%	2100 (61.0)	44 (57.9)	0.57
Heart failure	1270 (40.0)	42 (55.3)	0.0011
Peripheral arterial disease	433 (12.5)	21 (27.6)	0.0001
GFR (mL/min per 1.73 m^2^)			<0.0001
≥60	2348 (68.3)	23 (30.3)	
<60	1089 (31.7)	53 (70.0)	
Diabetes on medications	993 (28.9)	26 (34.2)	0.312
Anemia	1401 (40.8)	48 (63.2)	<0.0001
Emergent surgery	286 (8.3)	16 (21.1)	<0.0001
Preoperative shock	102 (3.0)	8 (11.0)	0.0002
Re-operative procedure	442 (12.9)	13 (17.1)	0.276
Intraoperative, *n* (%)			
Type of surgery			0.262
CABG only	1063 (30.9)	20 (26.3)	
Single Valve	419 (12.1)	6 (7.9)	
Combined Valve(s) ± CABG	1955 (56.9)	50 (65.8)	
Bypass duration, median (IQR), min	106 (83–140)	140 (96–185)	<0.0001
Nadir hematocrit on pump, *n* (%)			<0.0001
<0.21	73 (2.1)	5 (6.6)	
0.21–0.25	881 (25.6)	35 (46.0)	
>0.25	2443 (71.1)	35 (46.1)	
RBC transfusion, median (IQR), units	0 (0–1)	2 (1–5)	<0.0001
RBC transfusion of >4 units, *n* (%)	228 (6.6)	28 (36.8)	<0.0001
Vasopressor Pre-CPB	603 (17.5)	31 (40.8)	<0.0001
Vasopressor During CPB	1249 (36.3)	50 (65.8)	<0.0001
Vasopressor Post-CPB	1759 (51.2)	59 (77.6)	<0.0001
Inotrope Post-CPB	1047 (30.5)	43 (56.6)	<0.0001
Postoperative, *n* (%)			
New-onset atrial fibrillation	369 (10.7)	30 (39.5)	<0.0001

Abbreviations: IQR = interquartile range; LVEF = left ventricular ejection fraction; GFR = glomerular filtration rate; CABG = coronary artery bypass grafting; RBC = red blood cell; CPB = cardiopulmonary bypass.

**Table 2:** Baseline and perioperative characteristics of high-risk patients, as defined by a Cardiac Anesthesia Risk Evaluation score of 5.

Variable	Not Deceased	Deceased	*p*-Value
	(*n* = 306)	(*n* = 48)	
Demographic			
Age, median (IQR), year	61 (54–70)	63 (54–74)	<0.0001
Female sex, *n* (%)	80 (26%)	14 (29%)	0.659
Medical history, *n* (%)			
Hypertension	149 (48.7)	28 (58.3)	0.214
LVEF < 35%	224 (73.2)	29 (60.4)	0.068
Heart failure	236 (77.1)	36 (75.0)	0.746
Peripheral arterial disease	29 (9.5)	5 (10.4)	0.837
GFR (mL/min per 1.73 m^2^)			0.0065
≥60	179 (58.5)	18 (37.5)	
<60	127 (41.5)	30 (62.5)	
Diabetes on medications	62 (20.6)	10 (20.8)	0.969
Anemia	194 (63.4)	32 (66.7)	0.661
Emergent surgery	190 (62.1)	33 (68.8)	0.374
Preoperative shock	110 (35.9)	24 (50.0)	0.062
Re-operative procedure	69 (22.5)	16 (33.3)	0.104
Intraoperative, *n* (%)			
Type of surgery			0.33
CABG only	22 (7.2)	3 (6.3)	
Single Valve	24 (7.8)	1 (2.1)	
Combined Valve(s) ± CABG	260 (85.0)	44 (91.6)	
Bypass duration, median (IQR), min	147 (106–194)	183 (120–278)	<0.0001
Nadir hematocrit on pump, *n* (%)			0.043
<0.21	22 (7.3)	4 (8.5)	
0.21–0.25	106 (35.2)	25 (53.2)	
>0.25	173 (57.5)	18 (38.3)	
RBC transfusion, median (IQR), units	2 (0–4)	6 (3–8)	<0.0001
RBC transfusion of >4 units, *n* (%)	102 (33.3)	33 (68.8)	<0.0001
Vasopressor Pre-CPB	95 (31.4)	22 (45.8)	0.043
Vasopressor During CPB	169 (55.2)	31 (64.6)	0.224
Vasopressor Post-CPB	204 (66.7)	28 (58.3)	0.259
Inotrope Post-CPB	148 (48.4)	18 (37.5)	0.161
Postoperative, *n* (%)			
New-onset atrial fibrillation	76 (24.8)	17 (35.4)	0.122

Abbreviations: IQR = interquartile range; LVEF = left ventricular ejection fraction; GFR = glomerular filtration rate; CABG = coronary artery bypass grafting; RBC = red blood cell; CPB = cardiopulmonary bypass.

**Table 3:** Median durations of hypotension before, during and post cardiopulmonary bypass (CPB) and their corresponding morality risk in intermediate-risk patients. Absolute risk was defined as *n* (%) of patients who died after experiencing longer than median duration of hypotension at each of the Mean Arterial Pressure (MAP) thresholds.

	Duration of Hypotension (min), Median (IQR)			
Severity of Hypotension (mmHg)	Not Deceased (*n* = 3437)	Deceased (*n* = 76)	Absolute Risk, *n* (%)	*p*-Value
Pre CPB				
<55	2 (0–5)	3 (1–8)	40 (2.67)	0.074
55–64	10 (3–21)	18 (5–26)	47 (2.74)	0.022
During CPB				
<55	7 (3–15)	12 (6–24)	52 (3.03)	0.0006
55–64	24 (12–40)	42 (27–63)	60 (3.42)	<0.0001
Post CPB				
<55	1 (0–5)	9 (1–22)	53 (3.11)	0.0002
55–64	15 (6–28)	37 (11–60)	51 (3.00)	0.001

Abbreviations: CPB = cardiopulmonary bypass; IQR = interquartile range.

**Table 4:** Median durations of hypotension before, during and post cardiopulmonary bypass and their corresponding morality risk in high-risk patients.

	Duration of Hypotension (min), Median (IQR)			
Severity of Hypotension (mmHg)	Not Deceased (*n* = 306)	Deceased (*n* = 48)	Absolute Risk, *n* (%)	*p*-Value
Pre CPB				
<55	1 (0–6)	2 (0–18)	27 (15.7)	0.253
55–64	6 (0–17)	8 (0–25)	24 (13.6)	0.966
During CPB				
<55	10 (2–29)	8 (0–42)	22 (12.6)	0.621
55–64	26 (9–54)	19 (0–58)	20 (11.4)	0.23
Post CPB				
<55	1 (0–9)	2 (0–14)	25 (15.1)	0.414
55–64	16 (3–7)	21 (0–51)	28 (15.8)	0.214

Abbreviations: CPB = cardiopulmonary bypass; IQR = interquartile range.

**Table 5:** Adjusted odds ratio of in-hospital mortality, stratified by preoperative risk.

MAP (mmHg) Per 10 min	Intermediate-Risk Adjusted OR (95% CI)	*p*-Value	High-Risk Adjusted OR (95% CI)	*p*-Value
Pre-CPB				
<55	0.84 (0.63–1.13)	0.258	1.29 (0.91–1.82)	0.148
55–64	0.85 (0.71–1.02)	0.08	1.04 (0.90–1.21)	0.59
During CPB				
<55	0.97 (0.85–1.11)	0.659	0.97 (0.84–1.12)	0.65
55–64	1.10 (1.00–1.21)	0.049	1.01 (0.91–1.13)	0.798
Post-CPB				
<55	1.30 (1.13–1.49)	0.002	1.07 (0.97–1.18)	0.194
55–64	1.18 (1.07–1.30)	0.001	0.99 (0.88–1.10)	0.795

## Supplementary Materials

The following are available online at https://www.mdpi.com/2077-0383/9/7/2057/s1 Table S1: Baseline and perioperative characteristics of the overall cohort, Table S2: Multivariable predictors of in-hospital mortality in the overall cohort, Table S3a: Median durations of hypotension before, during and post cardiopulmonary bypass in intermediate-risk patients who developed postoperative complications vs. those who did not, Table S3b: Median durations of hypotension before, during and post cardiopulmonary bypass in high-risk patients who developed postoperative complications vs. those who did not, Table S4: Adjusted odds ratio of in-hospital mortality in patients with low preoperative risk, Table S5a: Multivariable predictors of in-hospital mortality in intermediate-risk patients, Table S5b: Multivariable predictors of in-hospital mortality in high-risk patients.

## Appendix A

**Table A1:** Covariates and their definitions.

Covariates	Definition
Hypertension	A. BP > 140 mmHg systolic or >90 mmHg diastolic in patients without diabetes or chronic kidney disease; or B. BP > 130 mmHg systolic or >80 mmHg diastolic on at least two occasions in patients with diabetes or chronic kidney disease; C. History of hypertension treated with medication, diet, and/or exercise
Congestive heart failure	Documented history of heart failure
Peripheral arterial disease	D. Claudication either with exertion or at rest; E. Amputation for arterial vascular insufficiency; F. Vascular reconstruction, bypass surgery, or percutaneous intervention to the extremities; documented abdominal aneurysm with or without repair; G. Positive noninvasive test (ankle brachial index 0.9, ultrasound, MRA, CTA of >50% in any peripheral artery) or angiographic imaging
Diabetes on medications	Diabetes mellitus treated with oral hypoglycemic and/or insulin
Anemia	Defined by the World Health Organization (<130 g/L for men and <120 g/L for women), based on the hemoglobin concentration measured closest to the time of surgery.
Emergent surgery	Surgery that must take place within 24 h of acute hospital admission
Preoperative shock	Requirement for inotropic support with evidence of end organ hypoperfusion or dysfunction or intraaortic balloon pump in situ before surgery
Intraoperative Transfusion	Number of packed red blood cells, expressed as a continuous variable
New-onset atrial fibrillation	New postoperative atrial fibrillation that was no present preoperatively

**Table A2:** Median durations of hypotension before, during and post cardiopulmonary bypass in intermediate-risk patients who developed postoperative complications vs. those who did not. Median durations of hypotension before, during and post cardiopulmonary bypass in high-risk patients who developed postoperative complications vs. those who did not.

**Duration of Hypotension (min), Median (IQR)**												
**MAP (mmHg)**	**Stroke**	**No Stroke**	***p*-Value**	**RRT**	**No RRT**	***p*-Value**	**Reopen**	**Not Reopened**	***p*-Value**	**Composite**	**None**	***p*-Value**
**Pre CPB**												
<55	3 (0–8)	2 (0–5)	0.036	2 (0–7)	2 (0–5)	0.020	2 (0–6)	2 (0–5)	0.953	2 (0–6)	2 (0–5)	0.003
55–64	15 (6–30)	10 (3–21)	0.007	14 (4–29)	10 (3–21)	0.0004	10 (2–23)	10 (3–21)	0.864	13 (4–27)	10 (3–21)	0.0008
**CPB**												
<55	15 (5–43)	7 (3–15)	<0.0001	12 (5–26)	7 (3–15)	<0.0001	7 (3–15)	7 (3–15)	0.003	11 (4–26)	7 (3–14)	<0.0001
55–64	34 (18–62)	24 (12–40)	0.0003	38 (16–60)	24 (12–39)	<0.0001	24 (12–40)	24 (12–40)	0.0003	33 (16–58)	23 (12–39)	<0.0001
**Post CPB**												
<55	5 (0–14)	1 (0–5)	0.0006	4 (0–14)	1 (0–5)	<0.0001	1 (0–5)	1 (0–5)	0.003	3 (0–11)	1(0–5)	<0.0001
55–64	23 (10–42)	15 (6–28)	0.0003	25 (10–48)	14 (6–27)	<0.0001	14 (6–28)	14 (6–28)	<0.0001	22 (9–41)	14 (6–26)	<0.0001
**Duration of Hypotension (min), Median (IQR)**												
**MAP (mmHg)**	**Stroke**	**No Stroke**	***p*-Value**	**RRT**	**No RRT**	***p*-Value**	**Reopen**	**Not Reopened**	***p*-Value**	**Composite**	**None**	***p*-Value**
**Pre CPB**												
<55	4 (0–18)	1 (0–6)	0.044	1 (0–7)	1 (0–6)	0.522	0 (0–2)	2 (0–8)	0.0002	1 (0–6)	2 (0–6)	0.165
55–64	6 (0–21)	6 (0–18)	0.771	6 (0–20)	7 (0–18)	0.837	3 (0–17)	7 (1–18)	0.187	6 (0–17)	7 (1–18)	0.486
**CPB**												
<55	26 (2–51)	9 (2–29)	0.076	12 (2–31)	9 (2–30)	0.666	9 (2–17)	11 (2–33)	0.271	12 (2–31)	9 (2–31)	0.791
55–64	44 (8–93)	25 (8–52)	0.074	25 (8–54)	25 (7–55)	0.974	26 (9–55)	25 (8–55)	0.718	25 (8–55)	26 (7–52)	0.872
**Post CPB**												
<55	5 (0–15)	1 (0–9)	0.076	3 (0–13)	0 (0–6)	0.0009	3 (0–10)	1 (0–9)	0.247	0 (0–6)	0 (0–6)	0.0003
55–64	21 (1–51)	16 (3–39)	0.514	22 (3–57)	15 (2–33)	0.003	25 (6–47)	15 (2–37)	0.047	21 (4–51)	14 (2–30)	0.004
