# Supplementary material for: The Impact of Preoperative Risk on the Association between Hypotension and Mortality after Cardiac Surgery: An Observational Study
Source: J Clin Med. 2020 Jun 30;9(7):2057. doi: 10.3390/jcm9072057 (PMC7408639; doi:10.3390/jcm9072057)
Supplement: Supplementary file 1 [file jcm-09-02057-s001.pdf]

Supplemental Table S1. Baseline and perioperative characteristics of the overall cohort.

Variable	Not Deceased	Deceased	P-value
	**(n = 6,496)**	**(n = 131)**	
**Demographic**			
Age, median (IQR), year	67 (59-74)	73 (64-79)	<0.0001
Female sex, n (%)	1,808 (30.5)	44 (35.5)	0.146
**Medical history, n (%)**			
Hypertension	4,750 (73.1)	106 (80.9)	0.046
LVEF < 35%	3,970 (61.0)	77 (58.8)	0.587
Heart failure	1,854 (28.5)	80 (61.1)	<0.0001
Peripheral arterial disease	692 (10.6)	27 (20.6)	0.0003
GFR (mL/min per 1.73 m2)			<0.0001
≥ 60	4,876 (75.1)	46 (35.1)	
< 60	1,620 (24.9)	85 (64.9)	
Diabetes on medications	1,725 (26.6)	37 (28.2)	0.663
Anemia	2,163 (33.3)	82 (62.6)	<0.0001
Emergent surgery	483 (7.4)	49 (37.4)	<0.0001
Preoperative shock	213 (3.3)	32 (24.4)	<0.0001
Re-operative procedure	511 (7.9)	29 (22.1)	<0.0001
**Intraoperative, n (%)**			
Type of surgery			<0.0001
CABG only	2,880 (44.3)	29 (22.1)	
Single Valve	1,170 (18)	7 (5.3)	
Combined Valve(s) ± CABG	2,446 (37.7)	95 (72.5)	
Bypass duration, median (IQR), min	94 (75-122)	146 (100-216)	<0.0001
Nadir hematocrit on pump, n (%)			<0.0001
< 0.21	123 (1.9)	10 (7.6)	
0.21 – 0.25	1,441 (22.2)	62 (47.3)	
> 0.25	4,932 (75.9)	59 (45)	
RBC transfusion, median (IQR), units	0 (0-1)	3 (1-6)	<0.0001
RBC transfusion of > 4 units, n (%)	228 (6.6)	28 (36.8)	<0.0001
Vasopressor Pre-CPB	832 (12.8)	55 (42)	<0.0001
Vasopressor During CPB	1,781 (27.4)	84 (64.1)	<0.0001
Vasopressor Post-CPB	2,589 (39.9)	92 (70.2)	<0.0001
Inotrope Post-CPB	1,471 (22.6)	63 (48.1)	<0.0001
**Postoperative, n (%)**			
New-onset atrial fibrillation	593 (9.1)	50 (38.2)	<0.0001

Abbreviations: IQR = interquartile range; LVEF = left ventricular ejection fraction; GFR = glomerular filtration rate; CABG = coronary artery bypass grafting; RBC = red blood cell; CPB = cardiopulmonary bypass

Supplemental Table S2: Multivariable predictors of in-hospital mortality in the overall cohort.

Variable	Adjusted Odds Ratio (95% CI)	P-value
**Demographic**		
Age	1.02 (1.00 - 1.05)	0.025
Female sex, n (%)	1.05 (0.69-1.60)	0.811
**Medical history**		
Hypertension	2.10 (1.21-12.1)	0.008
LVEF < 35%	0.89 (0.59-1.33)	0.555

Heart failure	1.37 (0.89-2.13)	0.157
Peripheral arterial disease	1.50 (0.92-2.45)	0.106
GFR (mL/min per 1.73 m^2)		<0.0001
≥ 60	Reference	
<60	2.45 (1.56-3.84)	
Diabetes on medications	1.00 (0.84-1.19)	0.976
Anemia	1.05 (0.69-1.61)	0.819
Emergent surgery	1.94 (1.13-3.34)	0.017
Preoperative shock	1.39 (0.75-2.56)	0.293
Re-operative procedure	1.30 (0.76-2.21)	0.335
**Intraoperative**		
Type of surgery		0.419
CABG only	Reference	
Single Valve	0.55 (0.22-1.37)	
Combined Valve(s) ± CABG	0.93 (0.51-1.69)	
Mean arterial pressure < 55 mmHg		
Pre-CPB	0.97 (0.81-1.17)	0.755
During CPB	0.97 (0.89-1.06)	0.468
Post-CPB	1.17 (1.08-1.27)	0.0002
Bypass duration > 120 min	2.09 (1.23-3.53)	0.006
Red blood cell transfusion of > 4 units	2.84 (1.81-4.46)	<0.0001
Vasopressor Pre-CPB	1.63 (1.04-2.56)	0.032
Vasopressor During CPB	1.43 (0.88-2.34)	0.153
Vasopressor Post-CPB	0.93 (0.56-1.55)	0.789
Inotrope Post-CPB	0.85 (0.55-1.31)	0.463
New-onset atrial fibrillation	2.40 (1.57-3.67)	<0.0001

Supplemental Table S3a: Median durations of hypotension before, during and post cardiopulmonary bypass in intermediate-risk patients who developed postoperative complications vs. those who did not.

MAP (mmHg)	Duration of Hypotension (min), median (IQR)											
	Stroke	No Stroke	P-value	RRT	No RRT	P-value	Exploration for Bleeding	No Exploration	P-value	Composite	None	P-value
**Pre CPB**												
<55	3 (0-8)	2 (0-5)	0.036	2 (0-7)	2 (0-5)	0.020	2 (0-6)	2 (0-5)	0.953	2 (0-6)	2 (0-5)	0.003
55-64	15 (6-30)	10 (3-21)	0.007	14 (4-29)	10 (3-21)	0.0004	10 (2-23)	10 (3-21)	0.864	13 (4-27)	10 (3-21)	0.0008
**CPB**												
<55	15 (5-43)	7 (3-15)	<0.0001	12 (5-26)	7 (3-15)	<0.0001	7 (3-15)	9 (4-21)	0.003	11 (4-26)	7 (3-14)	<0.0001
55-64	34 (18-62)	24 (12-40)	0.0003	38 (16-60)	24 (12-39)	<0.0001	24 (12-40)	30 (16-55)	0.0003	33 (16-58)	23 (12-39)	<0.0001
**Post CPB**												
<55	5 (0-14)	1 (0-5)	0.0006	4 (0-14)	1 (0-5)	<0.0001	1 (0-5)	2 (0-8)	0.003	3 (0-11)	1(0-5)	<0.0001
55-64	23 (10-42)	15 (6-28)	0.0003	25 (10-48)	14 (6-27)	<0.0001	14 (6-28)	20 (9-36)	<0.0001	22 (9-41)	14 (6-26)	<0.0001

Abbreviations: IQR = interquartile range; MAP = mean arterial pressure; CPB = cardiopulmonary bypass; RRT = new onset renal replacement therapy.

Supplemental Table S3b: Median durations of hypotension before, during and post cardiopulmonary bypass in high-risk patients who developed postoperative complications vs. those who did not.

MAP (mmHg)	Duration of Hypotension (min), median (IQR)											
	Stroke	No Stroke	P-value	RRT	No RRT	P-value	Exploration for Bleeding	No Exploration	P-value	Composite	None	P-value
**Pre CPB**												
<55	4 (0-18)	1 (0-6)	0.044	1 (0-7)	1 (0-6)	0.522	0 (0-2)	2 (0-8)	0.0002	1 (0-6)	2 (0-6)	0.165
55-64	6 (0-21)	6 (0-18)	0.771	6 (0-20)	7 (0-18)	0.837	3 (0-17)	7 (1-18)	0.187	6 (0-17)	7 (1-18)	0.486
**CPB**												
<55	26 (2-51)	9 (2-29)	0.076	12 (2-31)	9 (2-30)	0.666	9 (2-17)	11 (2-33)	0.271	12 (2-31)	9 (2-31)	0.791
55-64	44 (8-93)	25 (8-52)	0.074	25 (8-54)	25 (7-55)	0.974	26 (9-55)	25 (8-55)	0.718	25 (8-55)	26 (7-52)	0.872
**Post CPB**												
<55	5 (0-15)	1 (0-9)	0.076	3 (0-13)	0 (0-6)	0.0009	3 (0-10)	1 (0-9)	0.247	3 (0-12)	0 (0-6)	0.0003
55-64	21 (1-51)	16 (3-39)	0.514	22 (3-57)	15 (2-33)	0.003	25 (6-47)	15 (2-37)	0.047	21 (4-51)	14 (2-30)	0.004

Abbreviations: IQR = interquartile range; MAP = mean arterial pressure; CPB = cardiopulmonary bypass; RRT = new onset renal replacement therapy.

Supplemental Table S4. Adjusted odds ratio of in-hospital mortality in patients with low preoperative risk.

MAP (mmHg) per 10 min	Adjusted OR (95% CI)	P-value
Pre-CPB		
<55	2.12 (0.50-9.01)	0.310
55-64	0.99 (0.48-2.03)	0.970
During CPB		
<55	0.82 (0.41-1.64)	0.571
55-64	1.02 (0.65-1.58)	0.941
Post-CPB		
<55	1.45 (0.59-3.57)	0.416
55-64	1.03 (0.55-1.92)	0.923

Supplemental Table S5a. Multivariable predictors of in-hospital mortality in intermediate-risk patients.

Variable	Adjusted Odds Ratio (95% CI)	P-value
**Demographic**		
Age	1.05 (1.02 - 1.09)	0.002
Female sex, n (%)	0.90 (0.52-1.57)	0.72
**Medical history**		
Hypertension	4.13 (1.40-12.13)	0.01
LVEF < 35%	0.77 (0.46-1.30)	0.324
Heart failure	1.17 (0.69-1.98)	0.567
Peripheral arterial disease	1.93 (1.09-3.44)	0.025
GFR (mL/min per 1.73 m^2)		0.005
≥ 60	Reference	
<60	2.31 (1.128-4.17)	
Diabetes on medications	1.07 (0.87-1.33)	0.517
Anemia	1.12 (0.65-1.95)	0.681
Emergent surgery	2.19 (0.95-5.06)	0.065
Preoperative shock	1.32 (0.46-3.78)	0.61
Re-operative procedure	1.25 (0.60-2.62)	0.557
**Intraoperative**		
Type of surgery		0.823
CABG only	Reference	
Single Valve	0.87 (0.30-2.53)	
Combined Valve(s) ± CABG	0.79 (0.37-1.67)	
Mean arterial pressure < 55 mmHg		
Pre-CPB	0.84 (0.63-1.13)	0.258
During CPB	0.97 (0.85-1.11)	0.66
Post-CPB	1.3 (1.13-1.49)	0.0002
Bypass duration > 120 min	2.12 (1.09-4.12)	0.027
Red blood cell transfusion of > 4 units	2.47 (1.37-4.46)	0.003
Vasopressor Pre-CPB	1.64 (0.93-2.90)	0.091
Vasopressor During CPB	1.50 (0.81-2.78)	0.198
Vasopressor Post-CPB	0.96 (0.48-1.90)	0.895
Inotrope Post-CPB	1.39 (0.80-2.42)	0.245
New-onset atrial fibrillation	2.85 (1.65-4.9)	0.0002

Abbrevations: LVEF = left ventricular ejection fraction, GFR = glomerular filtration rate, CABG = coronary artery bypass grafting, CPB = cardiopulmonary bypass.

Supplemental Table S5b. Multivariable predictors of in-hospital mortality in high-risk patients.

Variable	Adjusted Odds Ratio (95% CI)	P-value
**Demographic**		
Age	0.99 (0.96-1.02)	0.323
Female sex	1.24 (0.55-2.82)	0.607
**Medical history**		
Hypertension	2.57 (1.13-5.82)	0.024
LVEF < 35%	0.73 (0.33-1.60)	0.427
Heart failure	1.45 (0.55-3.84)	0.458
Peripheral arterial disease	0.65 (0.21-2.03)	0.454
GFR (mL/min per 1.73 m^2)		0.04
≥ 60	Reference	
< 60	2.37 (1.04-5.37)	
Diabetes on medications	0.89 (0.63-1.26)	0.525
Anemia	0.79 (0.34-1.84)	0.586
Emergent surgery	1.35 (0.61-3.00)	0.455
Preoperative shock	1.49 (0.65-3.42)	0.342
Re-operative procedure	1.23 (0.53-2.87)	0.628
**Intraoperative**		
Type of surgery		0.41
CABG only	Reference	
Single Valve	0.47 (0.04-6.10)	
Combined Valve(s) ± CABG	1.78 (0.37-8.61)	
Mean arterial pressure < 55 mmHg		
Pre-CPB	1.29 (0.91-1.82)	0.14
During CPB	0.97 (0.84-1.12)	0.65
Post-CPB	1.07 (0.97-1.18)	0.194
Bypass duration > 120 min	1.09 (0.42-2.79)	0.866
Red blood cell transfusion of > 4 units	3.61 (1.62-8.03)	0.002
Vasopressor Pre-CPB	2.12 (0.87-5.16)	0.097
Vasopressor During CPB	1.93 (0.70-5.28)	0.202
Vasopressor Post-CPB	0.42 (0.16-1.15)	0.091
Inotrope Post-CPB	0.38 (0.17-0.89)	0.026
New-onset atrial fibrillation	1.35 (0.63-2.92)	0.441

Abbrevations: LVEF = left ventricular ejection fraction, GFR = glomerular filtration rate, CABG = coronary artery bypass grafting, CPB = cardiopulmonary bypass.
